# In Vivo Retention Quantification of Supramolecular Hydrogels Engineered for Cardiac Delivery

**DOI:** 10.1002/adhm.202001987

**Published:** 2021-02-15

**Authors:** Maaike J. G. Schotman, Marijn M. C. Peters, Gerard C. Krijger, Iris van Adrichem, Remmert de Roos, John L. M. Bemelmans, Maarten J. Pouderoijen, Martin G. T. A. Rutten, Klaus Neef, Steven A. J. Chamuleau, Patricia Y. W. Dankers

**Affiliations:** ^1^ Institute for Complex Molecular Systems Laboratory of Chemical Biology Department of Biomedical Engineering Eindhoven University of Technology Groene Loper 7 Eindhoven 5612 AZ The Netherlands; ^2^ Department of Cardiology Experimental Cardiology Laboratory UMC Utrecht Regenerative Medicine Centre University Medical Centre Utrecht University Utrecht Heidelberglaan 100 Utrecht 3584 CX The Netherlands; ^3^ Department of Nuclear Medicine University Medical Centre Utrecht Utrecht 3584 CX The Netherlands; ^4^ SyMO‐Chem Den Dolech 2 Eindhoven 5612 AZ The Netherlands; ^5^ Institute for Complex Molecular Systems Laboratory of Chemical Biology Laboratory for Cell and Tissue Engineering Department of Biomedical Engineering Eindhoven University of Technology Groene Loper 7 Eindhoven 5612 AZ The Netherlands

**Keywords:** cardiac injection, imaging, quantification retention, radioactive labeling, supramolecular hydrogels

## Abstract

Recent advances in the field of cardiac regeneration show great potential in the use of injectable hydrogels to reduce immediate flush‐out of injected factors, thereby increasing the effectiveness of the encapsulated drugs. To establish a relation between cardiac function and retention of the drug‐encapsulating hydrogel, a quantitative in vivo imaging method is required. Here, the supramolecular ureido‐pyrimidinone modified poly(ethylene glycol) (UPy‐PEG) material is developed into a bioactive hydrogel for radioactive imaging in a large animal model. A radioactive label is synthesized, being a ureido‐pyrimidinone moiety functionalized with a chelator (UPy‐DOTA) complexed with the radioactive isotope indium‐111 (UPy‐DOTA‐^111^In) that is mixed with the hydrogel. Additionally, bioactive and adhesive properties of the UPy‐PEG hydrogel are increased by supramolecular introduction of a UPy‐functionalized recombinant collagen type 1‐based material (UPy‐PEG‐RCPhC1). This method enables in vivo tracking of the nonbioactive and bioactive supramolecular hydrogels and quantification of hydrogel retention in a porcine heart. In a small pilot, cardiac retention values of 8% for UPy‐PEG and 16% for UPy‐PEG‐RCPhC1 hydrogel are observed 4 h postinjection. This work highlights the importance of retention quantification of hydrogels in vivo, where elucidation of hydrogel quantity at the target site is proposed to strongly influence efficacy of the intended therapy.

## Graphical abstract

Exact quantification of hydrogel retention and redistribution upon injection is of utmost importance for determining the effectiveness of cardiac therapeutics. Thus, a modular approach to radioactively label a supramolecular hydrogel is developed, which is verified by in vivo scintigraphic imaging in a porcine model after cardiac injection. This method enables drug dose optimization and easy visualization of possible off‐target effects.

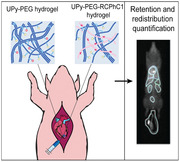

## 1 Introduction

Ischemic heart disease is responsible for over 9 million deaths per year worldwide as a result of blood flow deficiency in the cardiac area, resulting in adverse ventricular remodeling and contractile dysfunction.^[^ ^1^ ^]^ This adverse remodeling is caused by the inability of the heart to replace cardiomyocytes lost by ischemic damage to the myocardium. Therapeutic methods to stimulate cardiac repair remain ineffective as potential reparative drugs injected into the heart immediately flush‐out through the venous cardiac microvasculature and the injection needle tract.^[^ ^2^ ^]^ A carrier system to protect and localize regenerative factors at the injection site, and enable sustained, slow therapeutic release might provide a solution to this delivery issue. Biomaterials are increasingly studied in the field of cardiac regeneration, where patches or injectable hydrogels are applied to aid retention and provide sustained release of drug molecules at the target site.^[^ ^3^, ^4^ ^]^

Visualization of these biomaterials after injection or implantation is of high importance to assess the retention and degradation at the target site. Quantification of retention would enable correlation of drug efficacy to presence and availability at the target site, as well as monitoring the fate and distribution of the material in vivo. Based on this, volumes of injection or implantation could be tuned for an optimal drug release effect. Moreover, unwanted side effects at potential off‐target sites could be brought to light and prevented by obtaining an enhanced understanding of the distribution.

For retaining drugs at the site of injection, hydrogels are considered good candidates as biocompatible, easily injectable carrier systems for cardiac repair and regeneration.^[^ ^5^ ^]^ Previously, it was shown that the release of miR‐302 from a hyaluronic‐based hydrogel promoted cardiomyocyte proliferation and regeneration after myocardial infarction (MI) in a porcine heart.^[^ ^6^ ^]^ Furthermore, injection of a hydrogel based on decellularized extracellular matrix (ECM) in a rat MI model reduced cardiomyocyte apoptosis and enhanced neovascularization.^[^ ^7^ ^]^ Moreover, efficacy was established in a porcine infarction model^[^ ^8^ ^]^ and currently, this decellularized ECM‐based gel is in its first human‐trial to examine the safety and feasibility post MI.^[^ ^9^ ^]^ Delivery of these hydrogels is primarily performed via a minimally invasive catheter based injection technique, where gelation occurs at the target site triggered by temperature increase.^[^ ^10^ ^]^ While studying the beneficial effect of hydrogel‐mediated delivery of therapeutic factors, primarily indirect parameters (e.g., scar thickness, ejection fraction, and end‐diastolic volume) and the functional effect of the drugs were examined.^[^ ^11^, ^12^, ^13^ ^]^ Only a few studies examined the degree of hydrogel retention after injection and the potential off‐target distribution of the gel, mainly in small animal models.^[^ ^14^, ^15^, ^16^ ^]^ A collagen matrix delivery in a mouse model with MI was assessed on its retention and distribution by Positron Emission Tomography (PET) imaging, where the hydrogel was labeled with hexadecyl‐4‐[(18)F]fluorobenzoate ((18)F‐HFB).^[^ ^17^ ^]^ A more recent study showed in vivo nuclear imaging of an alginate hydrogel in which the nuclear imaging radio‐metal indium‐111 (^111^In) was incorporated.^[^ ^16^ ^]^ Intramyocardial injection in mice was performed, where a low retention was observed after 1 week (2–4%). To our knowledge, no study has provided quantitative numbers on the amount of cardiac retention and distribution of a supramolecular hydrogel in a large animal model.

**Figure 1:**
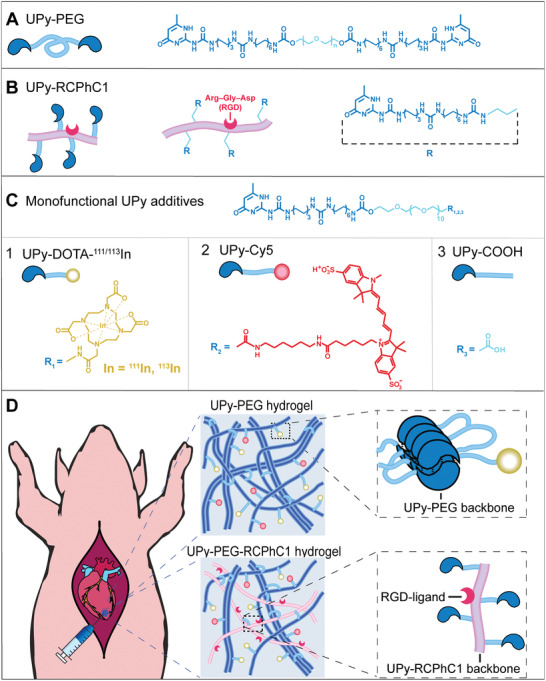
Hydrogel formulation and overview of this study. A,B) The chemical structure and schematic overview of hydrogelators A) UPy‐PEG and B) UPy‐PEG‐RCPhC1. C) The monofunctional UPy‐additives used during this study with the (non‐)radioactive label UPy‐^111/113^In 1), the fluorescently‐labeled UPy‐Cy5 2), and the nonfluorescent label mimic UPy‐COOH 3). D) The epicardial hydrogel injection in a porcine model, and the concept of the two different hydrogels used in this study, UPy‐PEG and UPy‐PEG‐RCPhC1.

Here, we show a first approach in performing a quantitative retention study by implementation of a radioactive tracer in our injectable pH‐ and temperature‐responsive supramolecular poly(ethylene glycol) (PEG) hydrogel functionalized at each end with ureido‐pyrimidinone (UPy) units (**Figure** **1**A).^[^ ^18^ ^]^ This UPy‐PEG hydrogelator is an injectable viscous liquid at a pH > 8.5 and rapidly forms a hydrogel once exposed to physiological pH.^[^ ^19^ ^]^ To increase tissue adhesiveness, a UPy‐functionalized recombinant peptide based on human collagen type 1 is introduced to the hydrogel (UPy‐PEG‐RCPhC1, Figure 1B), enriched with repeating amino acid sequences based on the integrin‐binding peptide arginine‐glycine‐aspartic acid (RGD). Supramolecular labeling of the hydrogel is performed using monofunctional UPy‐labels, for radioactive and for fluorescent visualization. The hydrogel is radioactively labeled using a 1,4,7,10‐tetraazacyclododecanetetraacetic acid (DOTA) chelated with radioactive isotope ^111^In, covalently bound to a monofunctional UPy‐moiety, allowing for in vivo radioactive detection. In addition, a fluorescent UPy‐Cy5 label was synthesized and used for ex vivo histological fluorescence analysis (Figure 1C). Comparative visualization and quantification of hydrogel retention and distribution was performed for epicardial injections of UPy‐PEG and UPy‐PEG‐RCPhC1 hydrogels in a porcine model (Figure 1D).

## 2 Results and Discussion

### 2.1 Molecules

**Figure 2:**
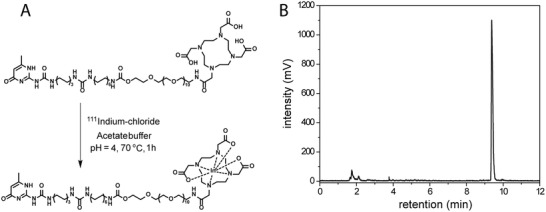
Chelation of the ^111^In with the UPy‐DOTA label, with A) the labeling method, and B) radio‐HPLC after chelation of UPy‐DOTA‐^111^In at basic pH (>9), with loose ^111^In at a retention time of 1.8–2.2 min and labeled UPy‐DOTA‐^111^In at 9.2–9.7 min, showing a chelation efficiency of 97%.

The UPy‐DOTA was chelated with the radioactive ^111^In label (**Figure** **2**A). Instant‐thin layer chromatography (iTLC) (SI Figure 1) as well as a separation and detection of radioactive compound by high‐performance liquid chromatography (radio‐HPLC) (Figure 2B) showed an optimal chelation after ≈1 h (with a varying range of 93–98%). The chelation remained stable at basic pH (Figure 2B) for ≈24 h. As a reference, a nonradioactive isotope indium‐113 (^113^In) was chelated to the UPy‐DOTA moiety (UPy‐DOTA‐^113^In) for in vitro measurements. A monofunctional UPy‐moiety functionalized with the fluorescent probe cyanine‐5‐amine (UPy‐Cy5), allowed histological staining after injection, where an unfunctionalized monofunctional UPy‐moiety (UPy‐COOH) was used as a nonfluorescent reference. To introduce bioactivity and potential adhesion to the cardiac tissue UPy‐RCPhC1 was added, synthesized by modification of pristine RCPhC1 with UPy‐units. ^1^H‐NMR spectroscopy showed an average number of six UPy‐moieties grafted on the RCPhC1 backbone (Figure S3, Supporting Information).

### 2.2 Hydrogel Preparation

The hydrogels were prepared in a simple mix‐and‐match manner, where the hydrogel precursors were dissolved at high pH Phosphate‐buffered saline ( PBS) (either UPy‐PEG, or UPy‐PEG in combination with UPy‐RCPhC1). Additives were introduced after the hydrogel precursors were fully dissolved. The molar ratio of the UPy‐PEG hydrogel for UPy‐PEG:UPy‐DOTA:UPy‐Cy5 or UPy‐COOH was set at 10:0.35:0.01. The additive ratio for UPy‐PEG‐RCPhC1 was identical, containing a 9:1 ratio of UPy‐PEG and UPy‐RCPhC1. The pH of the hydrogel precursor was adjusted to 9 or 8.8 ± 0.1 for UPy‐PEG and UPy‐PEG‐RCPhC1, respectively. The UPy‐PEG‐RCPhC1 hydrogelator shows similar viscosities at pH 8.8 to the UPy‐PEG hydrogelator at pH 9 (Figure S5, Supporting Information), which was desired to keep the flow properties similar.

### 2.3 Mechanical Properties of the Hydrogels

The myocardium shows viscoelastic characteristics, which is due to a combination of cardiac cells and ECM proteins. During heart failure, collagen accumulation can affect the viscoelasticity of the myocardium significantly.^[^ ^20^ ^]^ A hydrogel showing viscoelastic properties and accommodating the pulsating behavior of the heart is desired. To examine the mechanical properties of the hydrogels used in this study, UPy‐DOTA was chelated in acetate buffer with nonradioactive isotope ^113^In, where an optimal chelation efficiency was confirmed by RP LC‐MS (>95%, Figure S2, Supporting Information). A previous study reported that the release of a UPy‐DOTA label complexed with Gadolinium(III) from the UPy‐PEG hydrogel is in line with the rate of erosion of the hydrogel itself.^[^ ^21^ ^]^ Rheology was used to examine the influence of the additives (UPy‐DOTA‐^113^In and UPy‐COOH, Figure 1C3) on the mechanical properties of the hydrogels.

**Figure 3:**
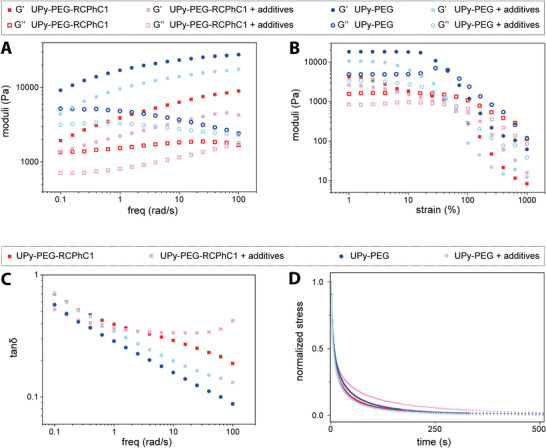
The mechanical properties of the hydrogels, showing A) the frequency sweep at 1% strain with frequencies between 0.1 and 100 rad s^−1^,B) the strain‐sweep at 1 rad s^−1^ with strains varying from 1% to 1000%, C) the tan *δ*, and D)the stress‐relaxation at 1% strain, measured over a time span of 500 s, showing viscoelastic properties of the UPy‐PEG, and the UPy‐PEG‐RCPhC1 hydrogel, measured at 37 °C.

After pH neutralization, the UPy‐PEG as well as the UPy‐PEG‐RCPhC1 hydrogels show frequency‐dependent viscoelastic behavior, i.e., an increase of G′, while frequency was increased, while G″ remains stable or decreases (**Figure** **3**A). This behavior is reflected in the tan *δ* (G″/G′) (Figure 3C), that showed a decrease for both hydrogels by a factor of 7, 5.3, 3.8, and 1.2 over the tested frequency range for the UPy‐PEG, UPy‐PEG with additives, UPy‐PEG‐RCPhC1, and UPy‐PEG‐RCPhC1 with additives, respectively. This indicates that the solid properties are getting more distinctive as the frequency is increased, whereas at lower frequencies more liquid‐like properties are observed. This suggests that at lower frequencies (longer measuring time) there is more time for structural rearrangement, i.e., characteristic interactions in the material can relax and the material starts to flow. At high frequencies (shorter measuring time) there is less time for rearrangement, i.e., all interactions remain present in the structure and have no time to relax, therefore displaying a less dynamic and more solid structure. For the UPy‐PEG hydrogel, the additives resulted in a weaker gel, at lower frequencies (with a G′ of 9.1 vs 4.4 kPa at 0.1 rad s^−1^, respectively), as well as higher frequencies (with a G′ of 27 vs 17 kPa at 100 rad s^−1^, respectively, Figure 3A, blue).

Previous work showed that addition of monofunctional UPy‐molecules to the bifunctional UPy‐PEG molecule showed to decrease the dynamics of the network, influencing the stiffness of this mixture.^[^ ^22^ ^]^ Addition of UPy‐DOTA and UPy‐COOH showed to decrease the stiffness, where the latter is added from a stock solution in dimethyl sulfoxide (DMSO), which caused a decrease in stiffness of the hydrogel. However, addition of the monofunctional UPy‐molecules show to increase the stiffness of the hydrogel as shown in previous studies, in comparison to solely DMSO addition to the hydrogel (Figure S6, Supporting Information). Both UPy‐PEG hydrogels showed similar frequency dependent behavior, with increased stiffness at higher frequencies, and comparable viscoelastic properties.

A significant decrease in moduli is observed for the UPy‐PEG‐RCPhC1 hydrogel, where the UPy‐PEG hydrogel showed to be a stiffer gel (2–3 times stiffer in comparison to the UPy‐PEG hydrogel). This could be explained by the lower number of UPy‐moieties in this hydrogel, decreasing the supramolecular cross‐linking ability and therefore the stiffness of the hydrogel. Comparison of UPy‐PEG‐RCPhC1 with and without additives showed small differences in moduli (Figure 3A, red). The hydrogel with additives displayed lower storage modulus (with a G′ of 1.9 vs 1.3 kPa at 0.1 rad s^−1^) and loss modulus (with a G″ of 1.4 vs 0.7 kPa at 0.1 rad s^−1^), indicating that the additives have an influence on the mechanical properties of the hydrogels. However, similar to the UPy‐PEG hydrogel, the frequency dependent viscoelastic behavior of both hydrogels is comparable. The stress‐relaxation measurements show similar relaxation curves for the UPy‐PEG hydrogel with and without additives, where 50% relaxation was achieved at 6.2 versus 8 s, respectively (Figure 3D, blue). For the UPy‐PEG‐RCPhC1 hydrogels with and without additives, only small differences can be observed, with a time of 6.8 versus 6.2 s at a 50% relaxation, respectively (Figure 3D, red). This indicates a minimum to no difference that is observed considering relaxation times. The UPy‐PEG hydrogel without additives shows a linear course for storage as well as loss moduli until a minimum of 25% deformation, whereas the UPy‐PEG hydrogel with additives shows a slight decrease in G′ and G″ after a strain of ≈5%. The UPy‐PEG hydrogels with and without additives are disrupted at 45% and 65% strain, respectively (Figure 3B, blue). A similar trend is observed for the UPy‐PEG‐RCPhC1 hydrogels with and without additives, where a decrease in G′ and G″ is observed after a strain of 6%. Here, disruption of the hydrogels with and without additives occur at ≈25% and 40% strain, respectively (Figure 3B, red). UPy‐PEG‐RCPhC1 hydrogels with and without additives showed small variability regarding the strain and frequency sweep, where differences in stiffness were observed (Figure S7, Supporting Information). This marks the variability of the UPy‐functionalized RCPhC1, introduced to the system.

Overall, minor differences in mechanical properties of hydrogel variants were observed between absolute moduli and stiffness of the hydrogels, which should not affect usability in envisioned applications.

### 2.4 In Vivo Injection in Pig Heart and Scintigraphy

For the porcine experiments, one day prior to in vivo injection the additives (UPy‐DOTA‐^111^In and UPy‐Cy5) were added to the dissolved hydrogel precursors. Per injection of 200 µL the obtained activity was 5–10 MBq. Detailed hydrogel formation can be found in the Experimental Section.

Hydrogels (UPy‐PEG or UPy‐PEG‐RCPhC1) were injected epicardially (6 × 200 µL) after thoracotomy into the left ventricular wall of beating porcine hearts (*n* = 2). Scintigraphic total body scans were performed 1, 2, 3, and 4 h after injections and biodistribution of the hydrogel was quantified as percentage of radioactive signal in each organ from total whole body radioactive signal to exclude remaining radioactive signal in the dead volume of the syringes. Areas with high radioactive signal are identified by bright red coloration and areas with low radioactivity are identified by blue coloration.

**Figure 4:**
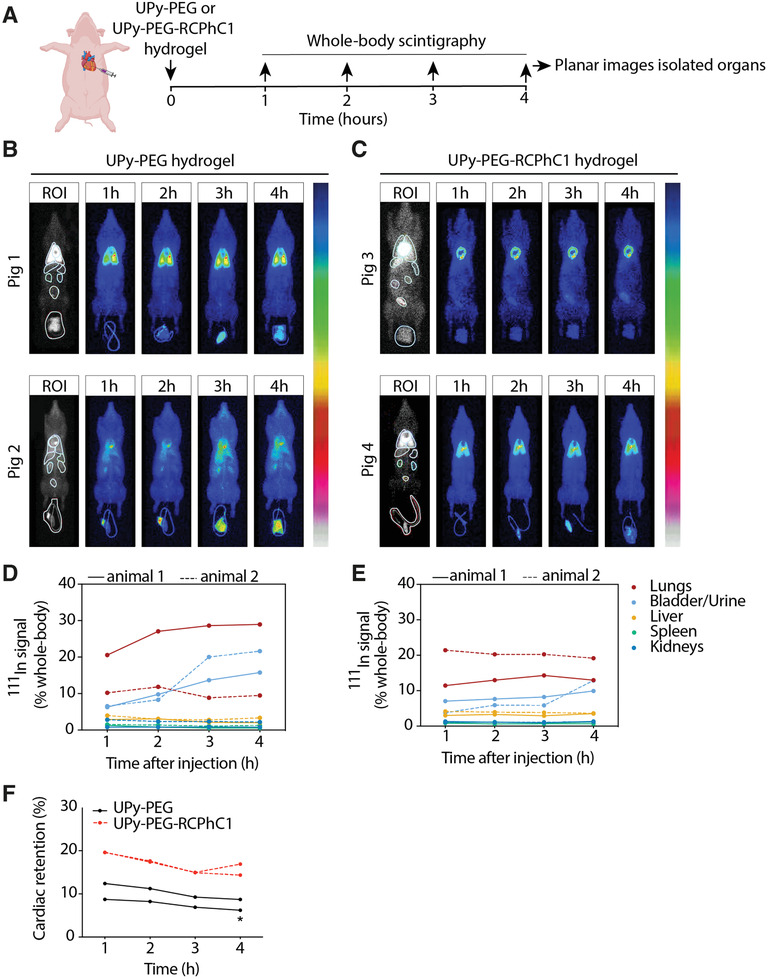
Scintigraphy imaging shows biodistribution of supramolecular hydrogels after epicardial injection. A) Experimental protocol. B,C) Whole‐body scintigraphy scans 1–4 h after injection of UPy‐PEG B) or UPy‐PEG‐RCPhC1 C) hydrogel. Areas with high radioactive signal are identified by bright red coloration and areas with low radioactivity are identified by blue coloration. D,E) Quantification of distribution in indicated remote organs after epicardial injection of UPy‐PEG D) or UPy‐PEG‐RCPhC1 E) hydrogel. F) Quantification of cardiac retention. Data are individual animals (*n* = 2 per hydrogel). Differences were evaluated using paired student's‐*t*‐test. * represents *P* < 0.05.

The total body scans showed that the injections of the UPy‐PEG led to a cardiac retention of 6.2% and 8.7% after 4 h (**Figure** **4**B,F). The remaining gel distributed to the lungs (29% and 9.5%), bladder and urine (15.8% and 21.6%), liver (2.0% and 3.4%), kidneys (0.8% and 2.2%), and spleen (0.56% and 1.28%) (Figure 4D). The residual activity was distributed evenly across the body without the presence of an increased and localized radioactive signal (“hotspots”).

Injection of UPy‐PEG‐RCPhC1 showed a cardiac retention of 16.9% and 14.3% after 4 h (Figure 4C,F). The remaining activity was distributed to the lungs (13.0% and 19.2%), bladder and urine (9.9% and 12.9%), liver (3.5% and 3.6%), kidneys (1.3% and 1.2%), and spleen (1.2% and 0.66%) (Figure 4C,E). Also, for the UPy‐PEG‐RCPhC1 the residual activity did not lead to hotspots. When comparing the cardiac retention of the two hydrogels at the 4 time points after injection, UPy‐PEG‐RCPhC1 injection showed increased retention (*P *< 0.05, *n* = 4).

The ^111^In signal in the lungs stabilized between 2 and 3 h after injection of the UPy‐PEG hydrogel in both pigs, while the ^111^In signal in the bladder and urine increased till 4 h after injection indicating secretion (Figure 4D). The UPy‐PEG‐RCPhC1‐hydrogel showed no increase in off‐target distribution after 1 h postinjection except for the ^111^In signal in the bladder and urine (Figure 4E). When comparing the off‐target biodistribution at the 4 timepoints after injection, no significant difference was found between the two hydrogels for lung biodistribution (*P *> 0.05, *α* = 0.19, *n* = 2) or bladder/urine biodistribution (*P *> 0.05, *α* = 0.09, *n* = 2).

During scintigraphic whole body scans, the measurement of radioactive signal from organs can be influenced by degree of tissue penetration and scattering, and by signals from overlapping organs. Therefore, 4 h after injection, organs were isolated and scanned individually.

**Figure 5:**
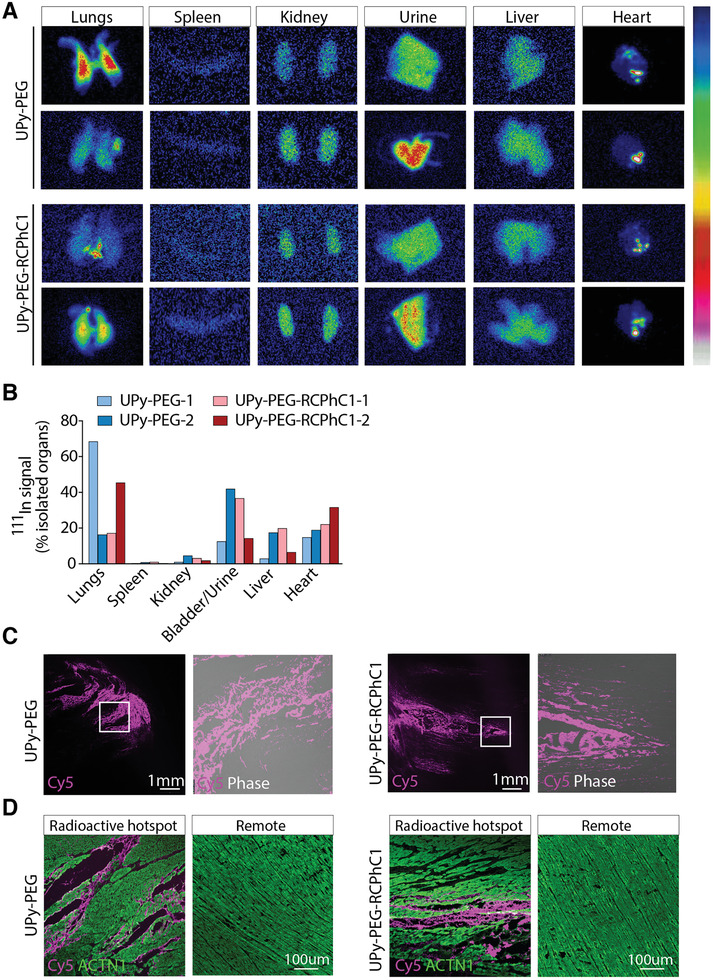
Scanning of isolated organs shows organ distribution of supramolecular hydrogels. A,B) Scintigraphic scans of individually isolated organs 4 h after epicardial injection of supramolecular hydrogels A) and quantification of radioactive signal as a percentage of total signal in the isolated main organs B). Areas with high radioactive signal are identified by bright red coloration and areas with low radioactivity are identified by blue coloration. C) Histology representative images of hydrogels by supramolecular bound fluorescent signal UPy‐Cy5 in the radioactive high intensity regions of the heart. D) Immunofluorescent staining of myocardium in radioactive hotspots and remote of radioactive hotspot. ACTN1, cardiac alpha actinin. Data are based on individual animals (*n* = 2 per hydrogel). Differences were evaluated using a paired student's *t*‐test.

Scanning of the explanted, isolated organs (heart, lungs, bladder/urine, liver, kidneys, spleen) indicated the retained radioactive signal in the heart was 14.8% and 18.9% for UPy‐PEG and 22.1% and 31.7% for UPy‐PEG‐RCPhC1 as a percentage of the total combined signal in the isolated organs (**Figure** **5**A,B). Furthermore, scintigraphy showed localized UPy‐PEG‐RCPhC1 at the individual injection sites with five to six radioactive hotspots (Figure 5A), corresponding to the individual injection sites. The UPy‐PEG injected pig hearts showed three to four more diffuse and merged injection sites. One UPy‐PEG injected heart showed increased radioactive signal at the base of the heart remote from the injection area (Figure 5A).

To verify presence of the gel at the sites with high radioactive signal in the heart, histological analysis was performed. The heart was sliced into five slices from apex to base and individual slices were scanned to identify sites of high ^111^In signal using a 1 Mbq point source (Figure S10, Supporting Information). These revealed intense Cy5 signal in sections of tissue with high radioactivity signal, showing deposits of seemingly condensed gel in larger interstitial spaces and strings of gel in the smaller interstitial spaces between individual cardiomyocytes (Figure 5C). This was further confirmed by immunofluorescent staining for cardiac alpha actinin (Figure 5D). During the washing steps of the staining procedure, the gel in the larger interstitial space dissolved, while the gel surrounding the cardiomyocytes was retained. At the areas remote from the radioactive high intensity regions, no Cy5 signal was found (Figure 5D). This pattern of distribution of the gel within the myocardium is hypothesized to be due to the flexibility of the gel to distribute and adhere to the myocardium and the extracellular matrix.

It is important to note only a small sample size of merely two animals per group was used to analyze in vivo hydrogel biodistribution. Therefore, no significant conclusions can be drawn on the difference between the two hydrogels in the in vivo experiments. However, the current study does give insights into the potential use of ^111^In labeling of injectable hydrogels for in vivo hydrogel retention quantification and comparison of retention and off‐target distribution of multiple hydrogels. When comparing the cardiac retention of UPy‐PEG and UPy‐PEG‐RCPhC1 in the four pigs we see a trend toward increased retention in the pigs injected with UPy‐PEG‐RCPhC1. This increase is likely to be caused by an increase in the adhesion of the gel to the extracellular matrix at the site of injection due to the integrin‐based recombinant peptide RCPhC1. Earlier studies using biomaterials to augment factor delivery, focused on the retention of delivered cells or compounds^,[^ ^12^ ^][^ ^18^ ^]^ or effects on functional parameters, rather than retention of the biomaterial.^[^ ^23^, ^24^ ^]^ Even though these studies gave insight into the potential benefits of hydrogel mediated delivery, so far no knowledge has been generated on the biodistribution of hydrogels in a large animal model after injection or quantitative approaches to determine the degree of retained hydrogel at the site of injection.

In addition, this shows a trend toward decreased off‐target distribution (lungs, bladder) and less diffuse distribution of UPy‐PEG‐RCPhC1 compared to UPy‐PEG hydrogel at the cardiac injection sites. However, we still report relatively high values of ^111^In signal in the lungs compared to other organs and intracardiac retention. When comparing our reported values of lung biodistribution to previous studies of labeled cells, we see that, of the small number of studies that report biodistribution after injection, similarly high levels of off‐target biodistribution to the lungs were reported. In previous studies in rats, the majority of injected cells were engrafted in the lungs 48 h after injection and cardiac retention values were reported below 1%.^[^ ^25^, ^26^ ^]^ Furthermore, a previous study in pigs showed labeled cell off‐target localization in the lungs of 26–47% with cardiac retention between 3% and 11% of the injected dose.^[^ ^27^ ^]^ Injection of an alginate hydrogel cross‐linked with radio‐metal ^111^In showed a cardiac retention of 2%–4%, one week postinjection.^[^ ^16^ ^]^

As a highly vascularized organ, it is likely this accumulation of ^111^In signal reflects dissolved UPy‐polymers that enter the lungs via the coronary veins or the central circulation. Previously, we have shown that the UPy‐polymers do not affect biocompatibility or induce toxicity.^[^ ^18^ ^]^ Further investigation into the effect of pulmonary biodistribution of intracardiac injected hydrogels is needed.

The distribution of the hydrogels to the lungs did not increase within 4 h after injection, considering the UPy‐PEG‐RCPhC1 hydrogel. The remaining off‐target distribution of the hydrogels to the bladder indicated clearance of the hydrogel through the secretory tract which limits the risk of extracardiac accumulation.

As the main cause of limited cardiac retention has been suggested to be the immediate clearing via the dense venous microvasculature and the needle track,^[^ ^2^ ^]^ we tracked the hydrogel biodistribution up to 4 h after injection. The limited changes within the presence of ^111^In signal in all organs within these 4 h is in line with previous studies on the importance to improve acute cardiac retention rather than chronic engraftment.^[^ ^27^, ^28^ ^]^

Even though the UPy‐based hydrogel gelates in physiological pH of the myocardium and shows increased retention compared to previously reported values, the presence of strong cardiac contractions and the needle track remains a challenge to maintain maximal cardiac retention of hydrogels. The achieved cardiac retention of ≈30% of UPy‐PEG‐RCPhC1 hydrogel is likely to increase effectiveness of regenerative therapies compared to nonhydrogel mediated delivery.

With the method described, we were able to quantitatively compare the retention of two supramolecular hydrogels and show these hydrogels appear to be safe on short term follow‐up of 4 h.

## 3 Conclusion

Quantitative imaging and distribution tracking after intramyocardial injection in a large animal model was achieved via modification of a pH‐switchable supramolecular hydrogel. In a simple mix‐and‐match manner, two types of hydrogels were radioactively labeled in a modular approach, with limited influence on the gelation properties. This ensured radioactive imaging of the hydrogels in a porcine heart in vivo to quantify and compare the retention of these supramolecular hydrogels. Introduction of integrin‐based recombinant peptide RCPhC1 to the hydrogel implied to increase hydrogel retention in the heart with defined deposits of hydrogel at the injection sites still visible after 4 h. Furthermore, the distribution of the hydrogels to other organs was reduced by addition of RCPhC1.

Finally, our method allows for dose quantification, increasing understanding of dose optimization and therefore drug effectiveness, which is of great importance in the translation toward cardiac regenerative therapy. Additionally, this method enables direct visualization of potential off‐target localization which can be used for efficient risk‐analysis of new therapies.

## 4 Experimental Section

### Materials

All starting materials and reagents were obtained from commercial sources and used as received, unless stated otherwise. Solvents from Sigma‐Aldrich were of p.a. quality. Deuterated chloroform and deuterium oxide were purchased at Cambridge Isotope Laboratories. Sulfo‐Cyanine5 amine was purchased at Lumiprobe. FujiFilm Manufacturing Europe B.V. kindly provided the Cellnest, a recombinant peptide based on human collagen type I (RCPhC1), which was used without further purification.

### Instrumentation

^1^H‐NMR spectra were recorded on a 400 MHz NMR operating at 400 MHz for functionalization analysis. Reverse‐phase high‐performance liquid chromatography‐mass spectrometry (RP‐HPLC‐MS) was performed on a Thermo scientific LCQ fleet spectrometer. The purity of UPy‐RCPhC1hC1 was determined with Waters Xevo G2 Quadrupole Time‐of‐Flight liquid chromatography–mass spectrometry equipped with an Agilent Polaris C18A reverse‐phase column (ID 2.0 mm, length 100 mm). Derivatives were dissolved in H_2_O (1 mg mL^−1^) and flowed (0.3 mL min^−1^) over the column using a 15–75% water/acetonitrile gradient with 0.1% formic acid prior to analysis in the positive mode in the mass spectrometer. Purification of UPy‐Cy5 was performed on a prep‐RP‐HPLC (using gradients of acetonitrile in water, with addition of 0.1 vol% trifluoroacetic acid), where collected fractions were freeze‐dried and analyzed by RP‐HPLC‐MS. Chelation efficiency was analyzed with two methods: instant thin layer chromatography with a glass microfiber chromatography paper impregnated with a silica gel stationary phase (Agilent Technologies) and sodium chloride 0.9% as a mobile phase, and high performance liquid chromatography (Thermo Scientific Dionex UltiMate 3000), both with NaI(Tl) detector for gamma rays (Canberra). A relatively geometry independent dose calibrator (VDC‐404, Veenstra Instruments, the Netherlands) was used to quantify the activity preinjection.

### Synthesis of UPy‐DOTA

The precursors UPy‐C_6_‐U‐C_12_‐C‐OEG_12_‐NH_2_ and N‐hydroxysuccinimide activated DOTA (DOTA‐NHS‐xTFA) were synthesized as described elsewhere.^[^ ^29^ ^]^ UPy‐C_6_‐U‐C_12_‐C‐OEG_12_‐NH_2_ (280 mg, 0.26 mmol) was dissolved in dimethylformamide (DMF, 5 mL) and DOTA‐NHS‐xTFA (383 mg, 0.53 mmol) and DiPEA (0.62 mL, 3.57 mmol) were added. The reaction mixture was stirred overnight and subsequently the solvent was removed under vacuum and twice coevaporated with toluene. Eluting over reversed phase C18 column with a gradient ACN/water of 5/95 to 80/20 afforded the intermediate UPy‐DOTA (360 mg, 94%) as a white powder after freeze‐drying.

^1^H NMR (400 MHz, CDCl3/CD3OD) *δ* 5.86 (s, 1H), 4.19 (t, J = 4.7 Hz, 2H), 3.65 (s, 44H), 3.53 (dt, J = 13.3, 6.0 Hz, 7H), 3.43–3.18 (m, 14H), 3.11 (q, J = 7.0 Hz, 13H), 2.25 (s, 3H), 1.58 (p, J = 6.8 Hz, 2H), 1.48 (p, J = 7.2 Hz, 6H), 1.37 (q, J = 5.9, 3.7 Hz, 4H), 1.34–1.17 (m, 16H) ppm. ^13^C NMR (101 MHz, D_2_O‐NaOD) *δ* 179.94, 179.72, 175.39, 173.12, 168.35, 162.77, 159.58, 157.85, 157.34, 156.42, 104.28, 71.99, 69.80, 69.52, 69.32, 68.98, 68.83, 63.74, 58.71, 58.37, 57.16, 50.45, 40.62, 39.78, 39.25, 38.69, 30.21, 30.06, 29.66, 29.48, 29.39, 29.17, 26.89, 26.72, 26.42, 22.64 ppm.

LC‐MS (ESI) Rt = 5.75 min, m/z calc for C_66_H_122_N_12_O_23_, 1451.8 Da; found 484.83 [M+3H]^3+^, 726.6 [M+2H]^2+^, 737.5 [M+Na+H]^2+^, 1452.4 [M+H]^+^, 1473.9 [M+Na]^+^.

### Synthesis of UPy‐Cy5

The synthesis of the UPy‐COOH has been described previously,^[^ ^29^ ^]^ UPy‐COOH (2.36 mg, 2.08 µmol) was dissolved in DMF (2 mL). *N*,*N*‐Diisopropylethylamine (2.15 mg, 16.6 µmol) was added and the solution was stirred at room temperature for 15 min. Sulfo‐Cy5‐NH_2_ (2 mg, 27.0 µmol) dissolved in DMF (3 mL) was added to the solution and stirred for 1 h at argon environment. H_2_O (containing 0.1 v/v% formic acid, 20 mL) was added to the solution and centrifugated (4 min, 3000 rpm) followed by decantation. Ultrapure water was added (20 mL) and the product was lyophilized. The compound was purified with preparative RP‐HPLC using a gradient of 40% ACN in H_2_O (both containing 0.1 v/v% formic acid). Lyophilization yielded pure UPy‐Cy5 (1.75 mg, 9.4 µmol, 45%) as a blue solid. This was dissolved in DMSO at 1 mg mL^−1^ and used from this stock solution.

ESI‐MS: m/z Calc. for C_91_H_149_N_11_O_25_S_2_ 1861.37; Obs. [M+3H]^3+^ 621.33, [M+2H]^2+^ 931.17, [M+H]^+^ 1861.75.

### Synthesis of Hydrogelators UPy‐PEG and UPy‐RCPhC1

The hydrogelator UPy‐PEG with *M*_n_, PEG = 10 kg mol^−1^, was synthesized by SyMO‐Chem BV, Eindhoven, The Netherlands.^[^ ^19^ ^]^ Briefly, the PEG was added to 1,1′‐carbonyldiimidazole (CDI) in dichloromethane, after which excess of CDI was removed by precipitation in diethyl ether. This was coupled to 1,10‐diaminodecane, followed by precipitation in diethyl ether. Solid UPy‐isocyanate was added to a solution of diamine terminated‐PEG in a mixture of 1:1 dichloromethane and chloroform. The UPy‐RCPhC1 was synthesized in a similar manner as described previously by Spaans et al.^[^ ^30^ ^]^ In short, UPy‐hexyl‐urea‐dodecyl‐amine was dissolved in DMSO and *N*,*N*‐diisopropylethylamine was added, whereafter CDI was added. The CDI functionalization was confirmed by RP‐HPLC‐MS, after which the solution was added to RCPhC1 dissolved in DMSO and left stirring overnight at argon environment.

### Preparation of Radioactively Labeled Hydrogel Precursor

Two days prior to in vivo injection 20 wt% hydrogel precursors were prepared by dissolving UPy‐PEG (36 µmol, 400 mg), or UPy‐RCPhC1 (2.53 µmol, 140 mg) and UPy‐PEG (23.2 µmol, 260 mg) in 1.6 mL basic PBS (pH 11.7, adjusted with 1 m NaOH) at 70 °C until fully dissolved after ≈1 h. After dissolving, the pH was adjusted to 9 and stored in the fridge until the following day. The following day, UPy‐DOTA compound (1.7 µmol, 2.5 mg) was dissolved at 2 mg mL^−1^ in acetate buffer (pH 4–5) at 50 °C for 30 min. The radioactive isotope indium‐111‐chloride (107 MBq, Curium, Petten, the Netherlands) was added to the UPy‐DOTA dissolved in acetate buffer. This was kept at 70 °C for 1 h, after which the chelation efficiency was examined. The chelation efficiency varied from 93 to 99%, determined by radio‐HPLC and iTLC. After chelation, the pH was adjusted to 9 using a 5 m, 1 m NaOH solution. This was then added to the hydrogel precursor solutions, where fluorescently labeled UPy‐Cy5 (25 nmol, 47 µg) from a DMSO stock solution (1 mg mL^−1^) was added .The weight percentage was adjusted to 10 wt% using basic PBS (pH 9). This was stored overnight in the fridge in the dark until injection the following morning. After loading of the six syringes (≈200 µL each), the exact activity per syringe was quantified using the dose calibrator.

### Preparation of Nonradioactively Labeled Hydrogel

One day prior to measuring, the hydrogel precursor solutions were prepared by dissolving UPy‐PEG (40 mg, 3.6 µmol), or (14 mg, 0.25 µmol), and UPy‐PEG (26 mg, 2.3 µmol) at 20 wt% in basic PBS (pH 11.7) at 70 °C until fully dissolved after ≈1 h. UPy‐DOTA compound (0.25 mg, 0.17 µmol) was dissolved in acetate buffer (2 mg mL^−1^, pH 4–5) at 50 °C for 30 min, after which indium‐^113^‐chloride (37.3 µg, 0.17 µmol) was added to the acetate buffer and kept at 70 °C for 1 h. The chelation was confirmed by RP‐LC‐MS. After chelation, the pH was adjusted to 9 using 5 m NaOH and 1 m NaOH solutions. This was added to the hydrogel precursor solution, where UPy‐COOH (4.7 µg, 2.5 nmol), the nonfluorescent reference of UPy‐Cy5, was added from a DMSO stock solution (1 mg mL^−1^). The hydrogel precursor solution was adjusted to pH 9, at a final weight percentage of 11 wt%. As control, UPy‐PEG (40 mg, 3.6 µmol), or (14 mg, 0.25 µmol), and UPy‐PEG (26 mg, 2.3 µmol) were dissolved in basic PBS (pH 11.7) at 70 °C until fully dissolved. The pH was adjusted to 9, and the final weight percentage was 11 wt%. This was stored in the fridge until used the following morning. For the rheological measurements, hydrogel disks were made in cylindrical Teflon molds (diameter of 8 mm, height of 2 mm). Precursor gels (100 µL, pH 9) were pipetted in the molds, where 10 µL of acidic PBS (10 µL, 13 × 10^−3^ m HCl) was added, resulting in a final weight percentage of 10 wt%. This was left to equilibrate for ≈1.5 h before measuring.

### Rheological Measurements

Rheological characterization of the hydrogels was performed on a discovery hybrid rheometer (DHR‐3, TA Instruments), using a flat stainless‐steel geometry with a diameter of 8 mm, with gap heights varying from 500 to 1000 µm. Low viscosity silicon oil (47 V 100, RHODORSIL) was used around the hydrogel to limit evaporation during measurements at 37 °C. Frequency sweep measurements were performed at *ω* = 0.1 to 100 rad s^−1^, at a strain of *γ* = 1%. Stress‐relaxation was performed at a strain of *γ* = 1%, over a time span of 500 s, where the first 1 s of measurement time was disregarded. The data were normalized using the highest stress generated from this point onward. Strain‐sweep measurements were performed at strains between *γ* = 1% and *γ* = 1000%, with a frequency of *ω* = 1 rad s^−1^. Time sweeps of 60 s were performed in between measurements (data not shown), with a strain of *γ* = 1% and a frequency of *ω* = 1 rad s^−1^.

### Animals and Surgical Procedure

Four Topigs Norsvin pigs (age ≈6 months, weight 60–65 kg) received care in accordance with the guide for the care and use of laboratory pigs prepared by the Institute of Laboratory Animals. Experiments were approved by the Animal Experimentation Committee of the Medicine Faculty of the Utrecht University, the Netherlands. Sedation was mediated by intramuscular infusion of ketamine (10 mg kg^−1^), midazolam (0.4 mg kg^−1^), atropine (0.05 mg kg^−1^), and intravenous injection of thiopental (4 mg kg^−1^) via the cannulated ear vein. General anaesthesia was maintained by continuous infusion of cist‐atracurium (0.1 mg kg^−1^ h^−1^), midazolam (0.4 mg kg^−1^ h^−1^), and sufentanil (2.5 µg kg^−1^ h^−1^) via the cannulated ear vein. A thoracotomy was performed to gain access to the epicardium and the apex was loosely fixed with a Starfish Cardiac Positioner (Medtronic, Minneapolis, MN). Six hydrogel injections, with a total volume of 1.2 mL, were performed through a 25‐gauge needle. The thorax was closed prior to the first scintigraphic scan.

### Short‐Term In Vivo Tracking and Quantification

To detect the ^111^In label serial anterior and posterior total body scans were performed with a full field of view gamma scintillation camera with a medium energy general purpose (MEGP) collimator (Philips Skylight Gamma Camera System Dual SPECT with Philips JETStream Workplace R3.0). Photopeak windows of 20% were set at 174–274 keV. With a scan speed of 10 cm min^−1^ total body scans were made with a matrix of 512 × 1024. For static scans of the isolated organs a scan time of 300 seconds per scan was used and a matrix of 256 × 256. The images were quantified by identifying the heart, lungs, liver, spleen, kidneys and bladder in the anterior and posterior cumulative 174 and 274 keV images as 2D regions of interest (ROI) and determining the total number of counts and pixels per ROI. The square root of the total number of counts from the ROI obtained from the anterior total body scan and the ROI obtained from the posterior total body scan determined the geometric mean of the counts as described by Stratton et al.^[^ ^31^ ^]^ The geometric mean of the total body ROI was identified as the 100% value to determine the % uptake of the ^111^In signal in each organ.

### Histological Analysis

For immunostaining, hearts were snap frozen and cut into 10 µm sections. Fixation of the tissue was mediated by incubation in 100% Acetone for 10 min at 4 °C for 10 min before permeabilization for 10 min in 1% Triton X‐100 in PBS. Heart tissue was then blocked for 1 h in 10% normal whole goat serum (Vector laboratories S‐1000‐20) in PBS. Sections were stained overnight at 4 °C with sarcomeric *α*‐actinin (Sigma‐Aldrich A7811) after which 1 h incubation with secondary antibody conjugated with Alexa Fluor 488 in combination with nuclear marker Hoechst (Life Technologies 33 342) was performed. Sections were then mounted in Fluoromount‐GTM (ThermoFischer Scientific 00‐4958‐02) before imaging with SP8x Leica confocal microscope.

### Statistical Analysis

The in vivo data are expressed as individual animals as the sample size was *n* = 2 per group. Statistical analysis was performed per group in which the individual datapoints per scan were compared and evaluated with a paired student's *t*‐test to compare the biodistribution values at the different timepoints past injection per hydrogel. Data analysis was performed using GraphPad Prism version 8.0.0 for Mac OS X, GraphPad Software, San Diego, CA, www.graphpad.com. *P *< 0.05 was considered statistically significant.

## Conflict of Interest

The authors declare no conflict of interest.

## Supporting information

Supporting Information

## Data Availability

Research data are not shared.
