# Supplementary material for: In Vivo Retention Quantification of Supramolecular Hydrogels Engineered for Cardiac Delivery
Source: Adv Healthc Mater. 2021 Feb 15;10(10):2001987. doi: 10.1002/adhm.202001987 (PMC11468640; doi:10.1002/adhm.202001987)
Supplement: Supplementary file 1 — Supporting Information [file ADHM-10-2001987-s001.pdf]

© 2021 Wiley-VCH GmbH.

ADVANCED
HEALTHCARE
MATERIALS

Supporting Information

for *Adv. Healthcare Mater.,* DOI: 10.1002/adhm.202001987

In Vivo Retention Quantification of Supramolecular Hydrogels Engineered for Cardiac Delivery

*Maaike J.G. Schotman, Marijn M.C. Peters, Gerard C. Krijger, Iris van Adrichem, Remmert de Roos, John L. M. Bemelmans, Maarten J. Pouderoijen, Martin G. T. A. Rutten, Klaus Neef, Steven A.J. Chamuleau, Patricia Y.W. Dankers**

Supporting Information

In Vivo Retention Quantification of Supramolecular Hydrogels Engineered for Cardiac Delivery

*Maaike J.G. Schotman, Marijn M.C. Peters, Gerard C. Krijger, Iris van Adrichem, Remmert de Roos, John L. M. Bemelmans, Maarten J. Pouderoijen, Martin G. T. A. Rutten, Klaus Neef, Steven A.J. Chamuleau, Patricia Y.W. Dankers**

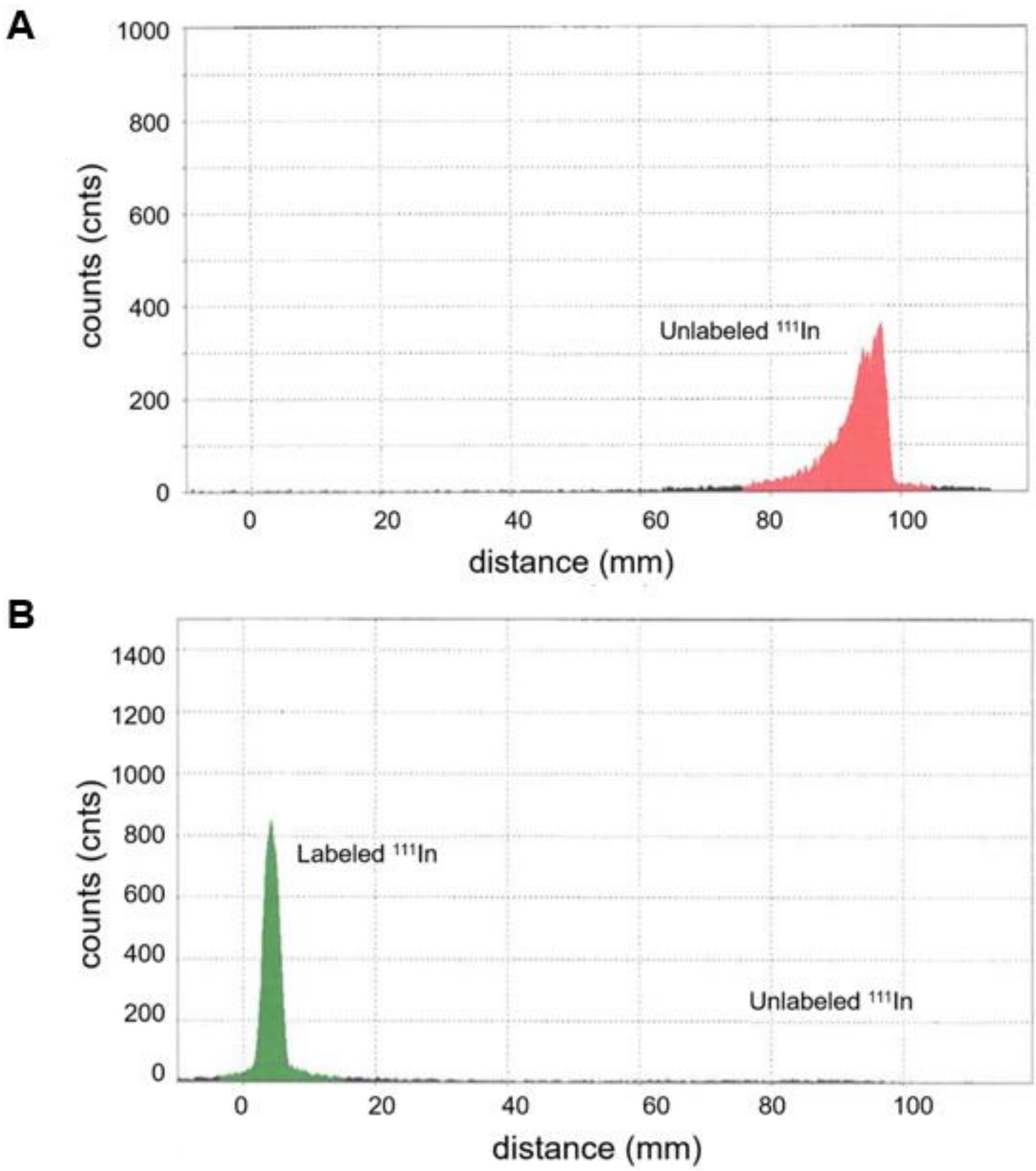

SI Figure 1. iTLC of the chelation efficiency of the UPy-DOTA with ^{111}In, with (A) the unchelated loose ^{111}In depicted in red, and (B) the chelated UPy-DOTA-^{111}In depicted in green, showing a chelation efficiency of approximately 99%.

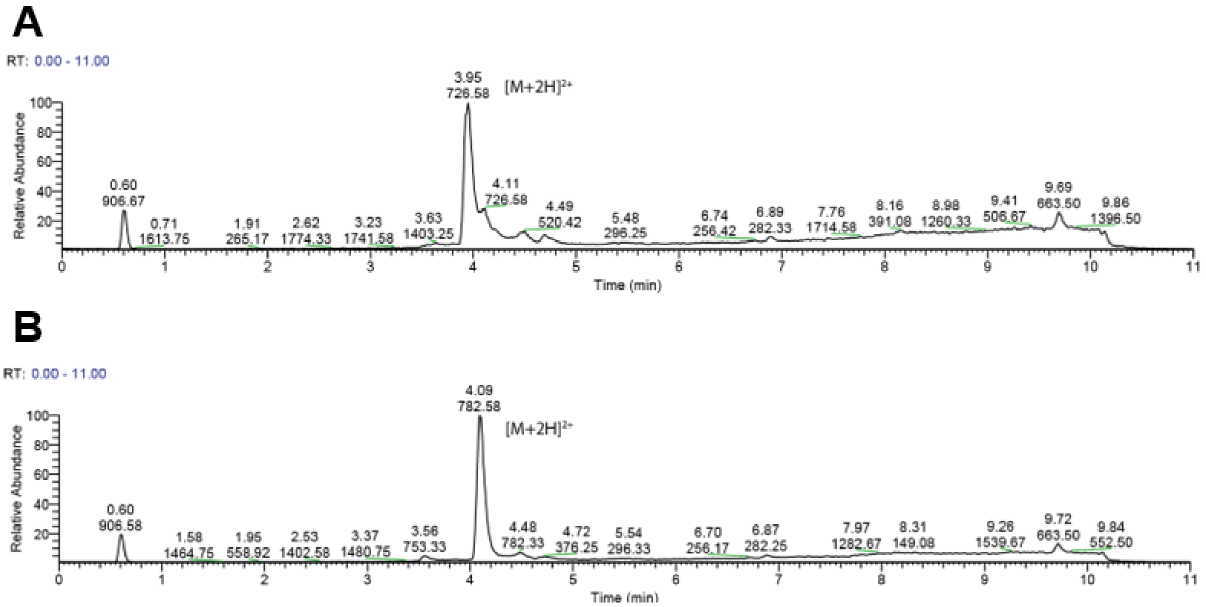

SI Figure 2. LC-MS spectra showing chelation efficiency of UPy-DOTA with non-radioactive isotope ^{113}In, with (A) the non-chelated UPy-DOTA at a retention time of 3.95 min, showing the m/z $[M+2H]^{2+}$=726.58 and (B) the chelated UPy-DOTA-^{113}In showing the m/z $[M+2H]^{2+}$=782.58

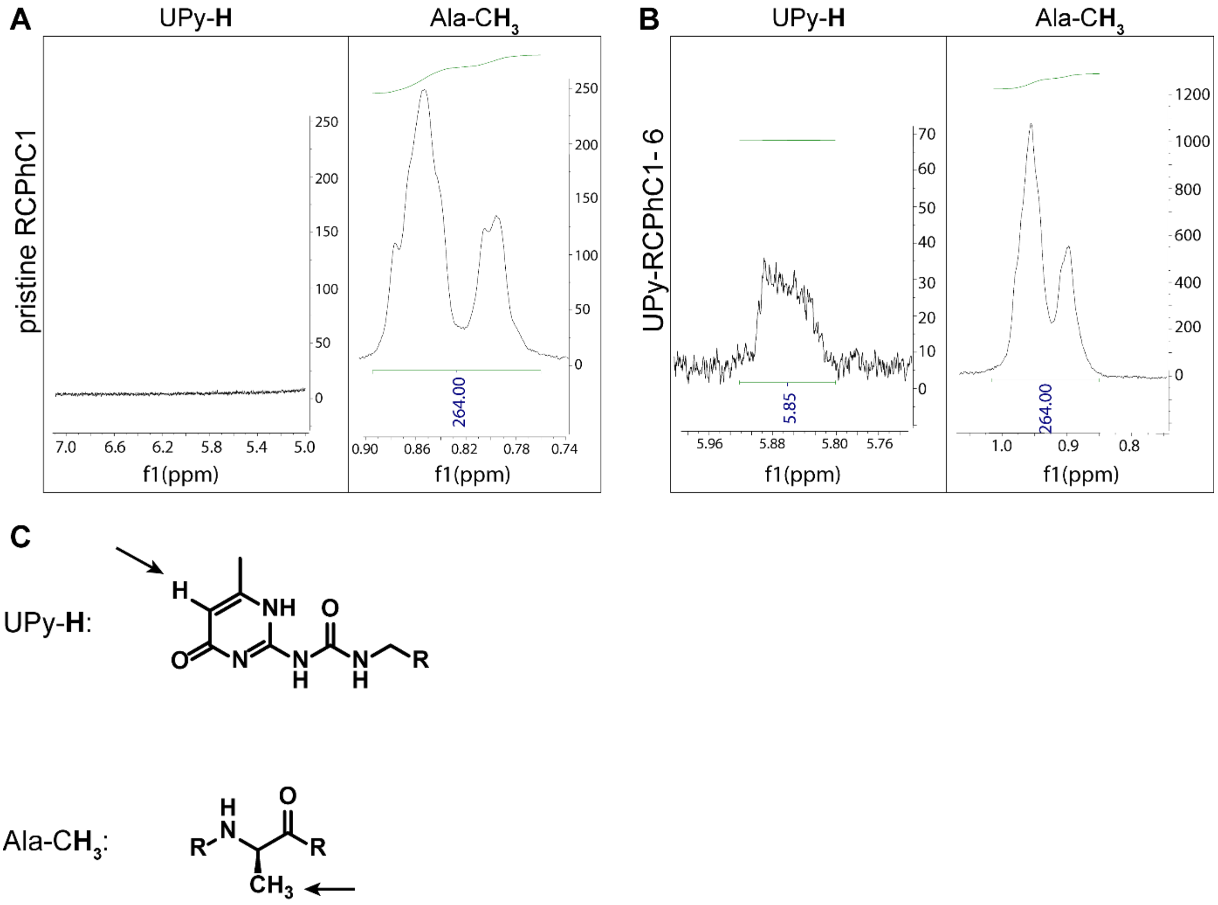

SI figure 3. Analysis of pristine recombinant collagen peptide derivative, modified with UPy-moieties. (A) ^{1}H NMR graph of RCPhC1 showing the protons of the alanine residues (δ-shift = 0.7 – 1.0), used as a reference, and the absent peaks of the alkylidene proton of the UPy-moiety (δ-shift = 5.8 – 5.9) for RCPhC1 and (B) UPy-RCPhC1 functionalized with an average of six UPy-moieties. (C) Schematic representation of the protons used to determine UPy-functionalization, with the alkylidene proton of the UPy-moiety and the methyl group on the alanine.

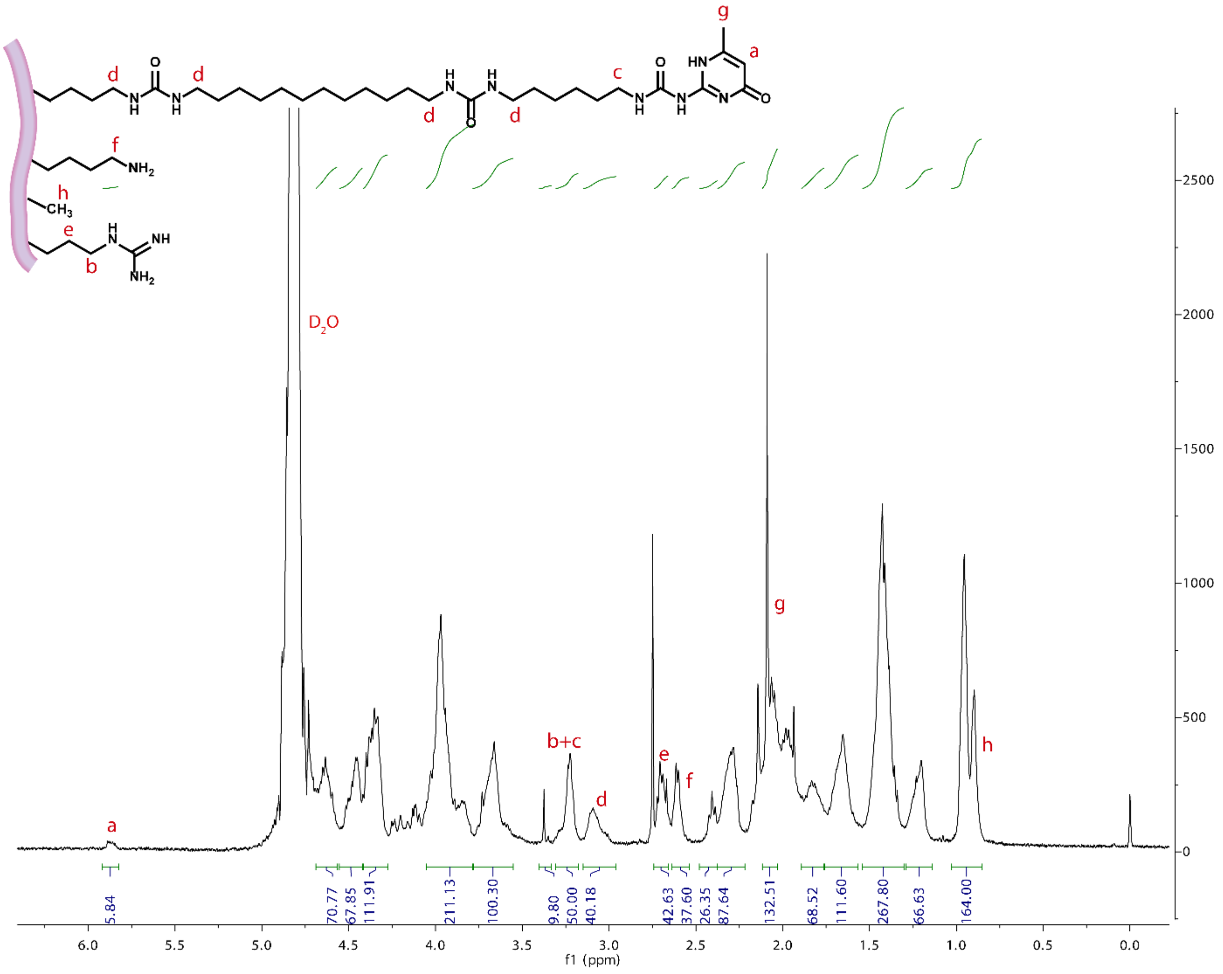

SI figure 4. ^{1}H-NMR of RCPhC1 functionalized with UPy-moieties showing relevant integration of protons that are part of lysine, alanine, arginine, and UPy residues.

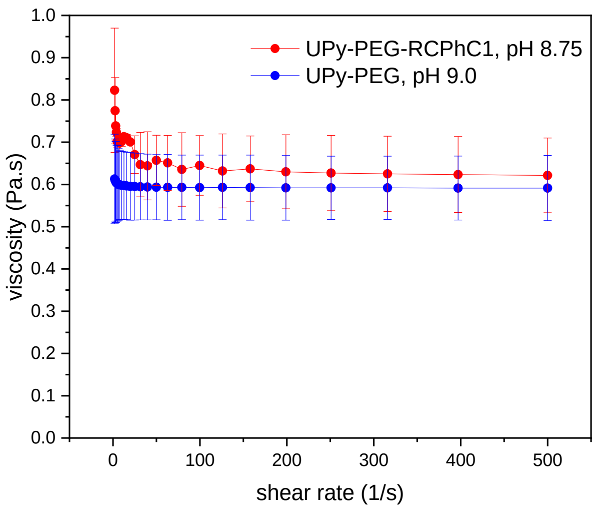

SI Figure 5. Viscosity of UPy-PEG-RCPhC1 and UPy-PEG at a pH of 8.75 and 9.0, respectively, showing similar viscosities at the different pH.

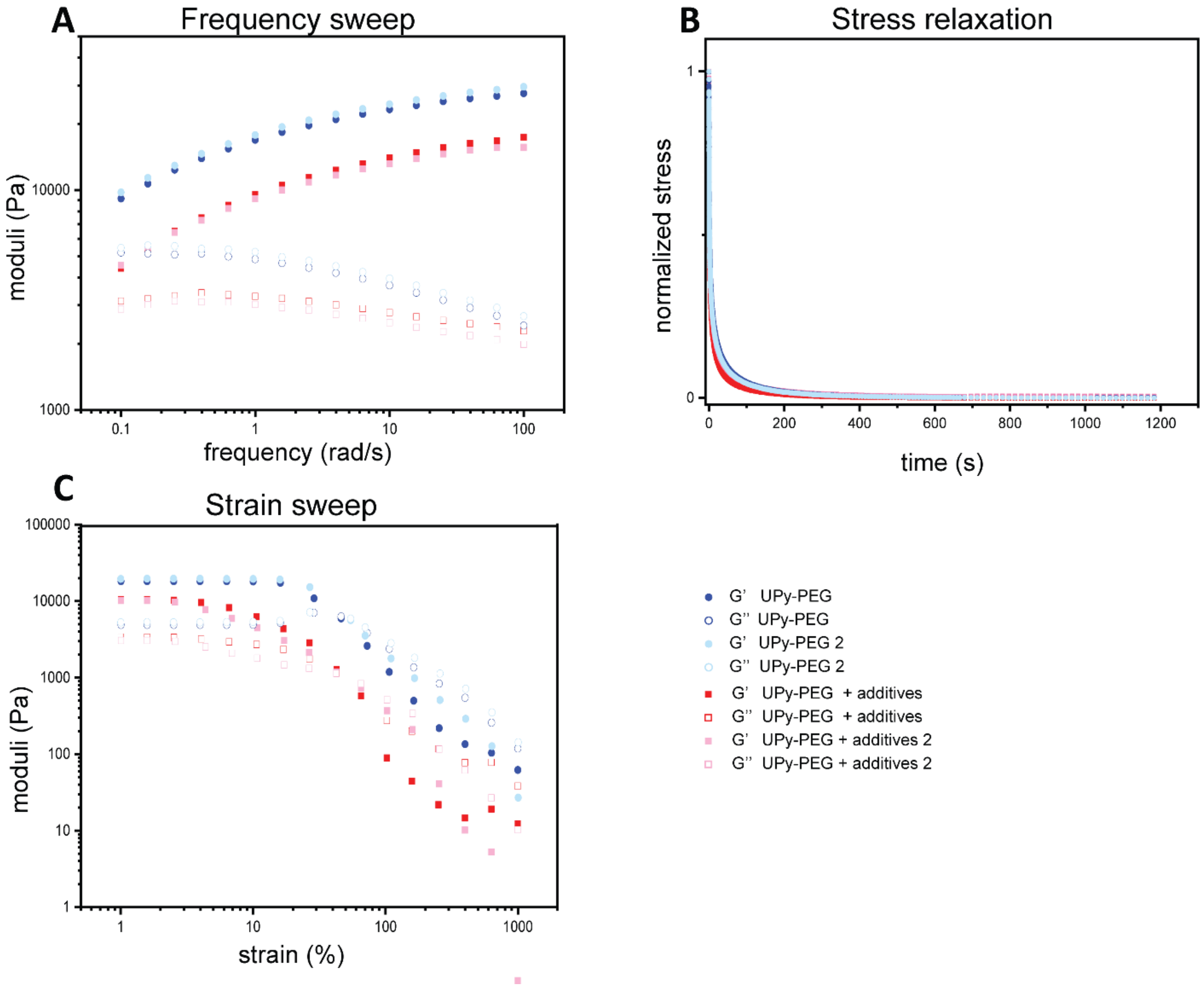

SI Figure 6. Mechanical properties of UPy-PEG with and without additives (*n*=2), with (A) the frequency-sweep at 1% strain, (B) the stress-relaxation at 1% strain, and (C) the strain-sweep at 1 rad.s^{-1} measured at 37 °C.

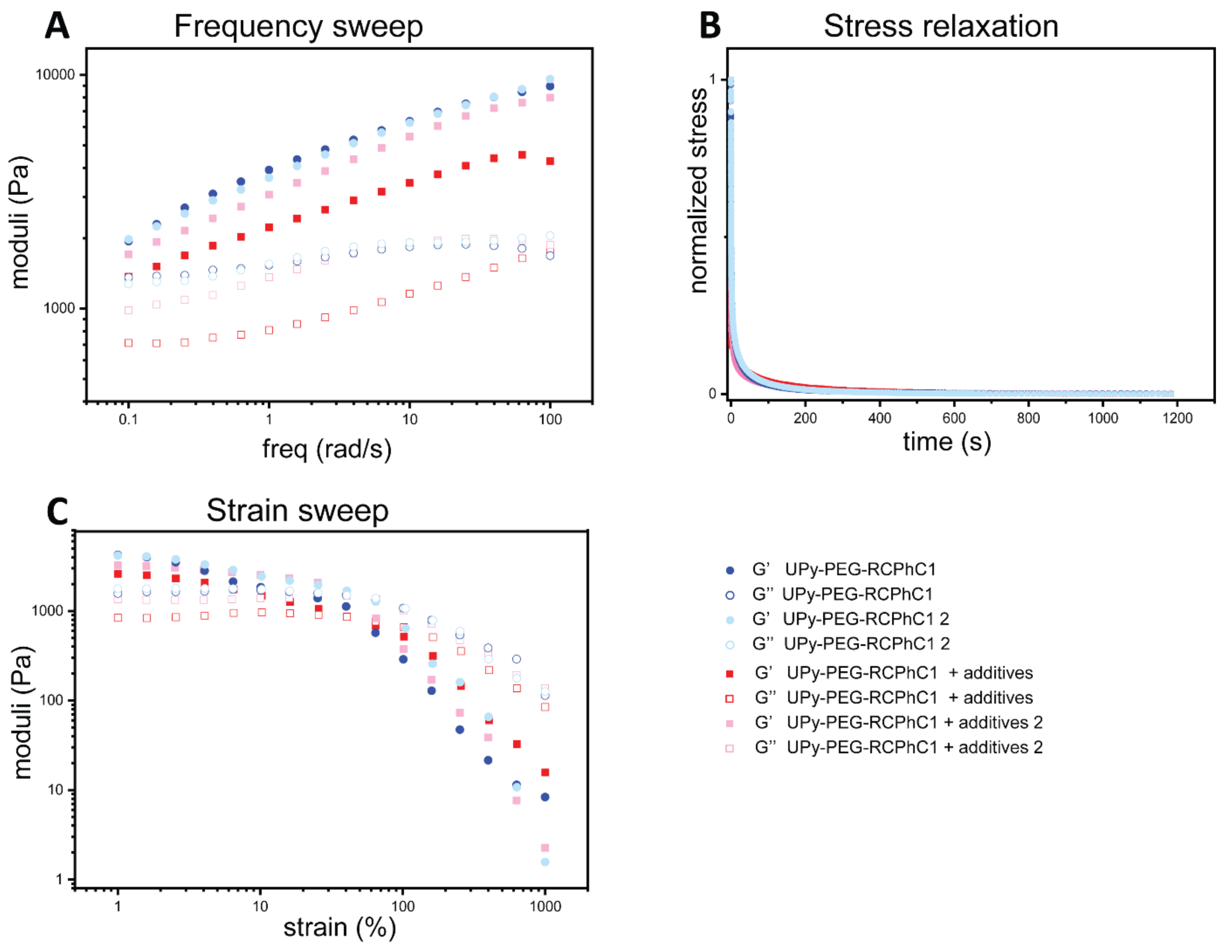

SI Figure 7. Mechanical properties of UPy-PEG-RCPhC1 with and without additives (*n*=2), with (A) the frequency-sweep at 1% strain, (B) the stress-relaxation at 1% strain, and (C) the strain-sweep at 1 rad.s^{-1} measured at 37 °C.

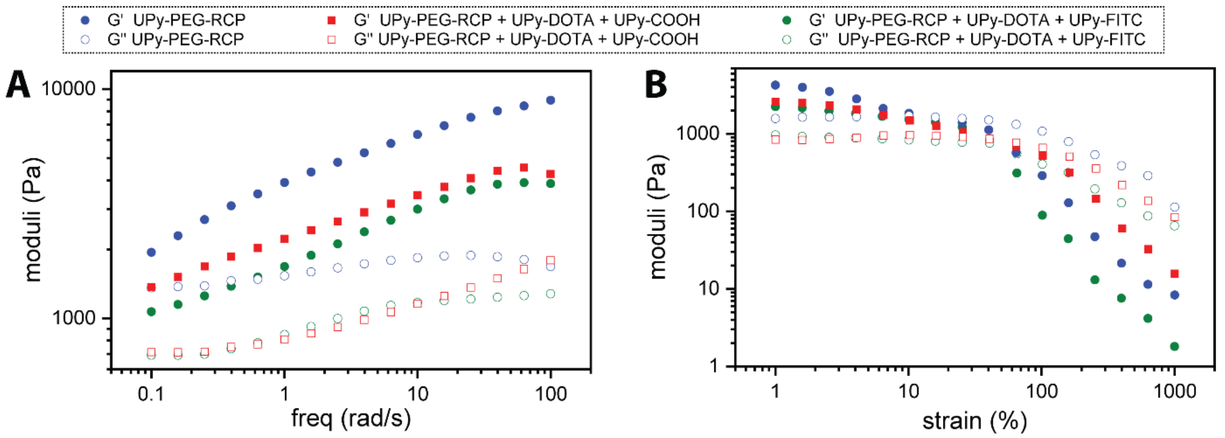

SI Figure 8. Mechanical properties of UPy-PEG-RCPhC1 upon addition of additives UPy-DOTA + UPy-COOH, and UPy-DOTA + UPy-FITC, with (A) the frequency-sweep at 1% strain, (B) the stress-relaxation at 1% strain, and (C) the strain-sweep at 1 rad.s^{-1} measured at 37 °C.

-1

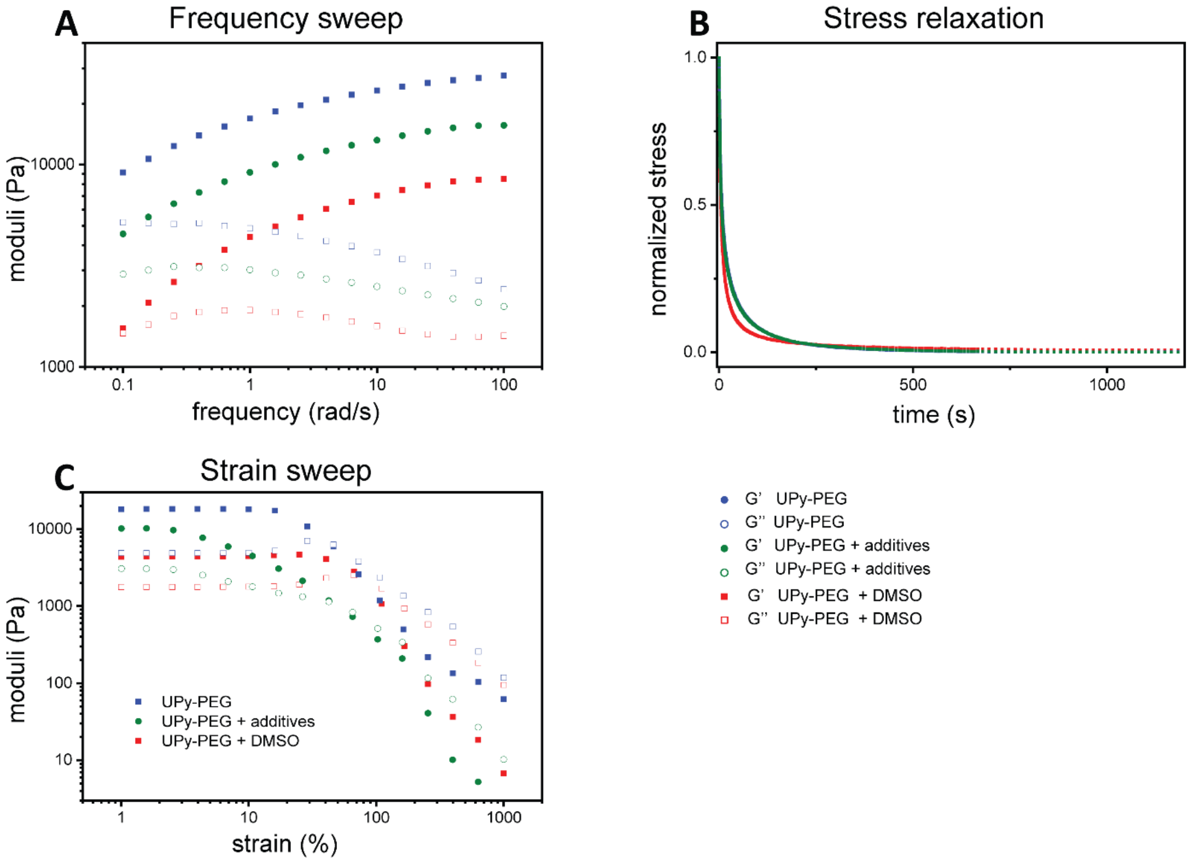

SI Figure 9. Mechanical properties of UPy-PEG with addition of all additives and DMSO, with (A) the frequency-sweep at 1% strain, (B) the stress-relaxation at 1% strain, and (C) the strain-sweep at 1 rad.s^{-1} measured at 37 °C.

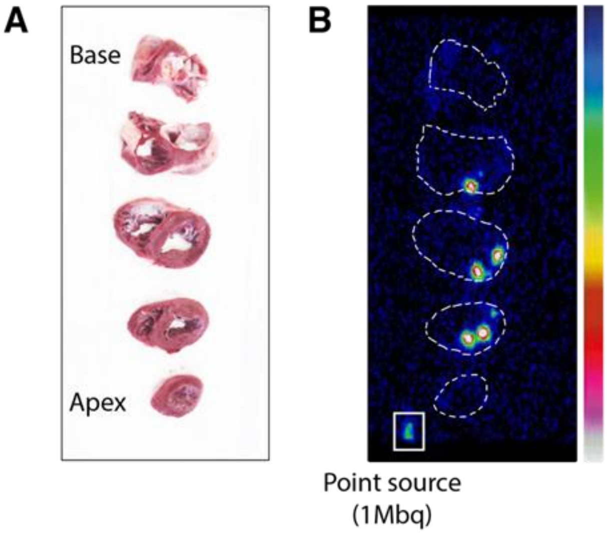

SI Figure 10. (A) Brightfield photograph of cardiac slices to identify exact sites of radioactive signal. (B) Scintigraphic scan of cardiac slices shown in A. A 1 Mbq point source was used to localize the exact part of tissue containing the ^{111}In signal.
